# Adaptive Thresholding Technique for Retinal Vessel Segmentation Based on GLCM-Energy Information

**DOI:** 10.1155/2015/597475

**Published:** 2015-02-24

**Authors:** Temitope Mapayi, Serestina Viriri, Jules-Raymond Tapamo

**Affiliations:** ^1^School of Mathematics, Statistics & Computer Science, University of KwaZulu-Natal, Durban 4000, South Africa; ^2^School of Engineering, University of KwaZulu-Natal, Durban 4041, South Africa

## Abstract

Although retinal vessel segmentation has been extensively researched, a robust and time efficient segmentation method is highly needed. This paper presents a local adaptive thresholding technique based on gray level cooccurrence matrix- (GLCM-) energy information for retinal vessel segmentation. Different thresholds were computed using GLCM-energy information. An experimental evaluation on DRIVE database using the grayscale intensity and Green Channel of the retinal image demonstrates the high performance of the proposed local adaptive thresholding technique. The maximum average accuracy rates of 0.9511 and 0.9510 with maximum average sensitivity rates of 0.7650 and 0.7641 were achieved on DRIVE and STARE databases, respectively. When compared to the widely previously used techniques on the databases, the proposed adaptive thresholding technique is time efficient with a higher average sensitivity and average accuracy rates in the same range of very good specificity.

## 1. Introduction

Retinal fundus imaging in ophthalmology is of great use in medical diagnosis and progression monitoring of several diseases like hypertension, diabetes, stroke, and cardiovascular disease [1]. Automatic vessel segmentation has a great potential to assist in the reduction of the time required by physicians or skilled technicians for manual labeling of retinal vessels [2].

Several retinal vessel segmentation techniques have been proposed and evaluated in literatures. Chaudhuri et al. [3] implemented a two-dimensional matched filter using a Gaussian shaped curve. However, the technique proposed in [3] achieved very low average accuracy due to low detection of retinal vessels. Hoover [4] segmented retinal vessels by applying a threshold probing technique combining local vessel attributes with region-based attributes on matched filter response (MFR) image. When compared to [3] where a basic thresholding of an MFR was used, the method proposed by [1] reduced the false positive rate by as much as 15 times. Fraz et al. [5] implemented vessel segmentation technique utilizing extracted center-lines of retinal vessels through first-order derivative of Gaussian filter. The authors used morphological operator with directional structuring elements to enhance the structure of blood vessels. They further generated the shape and orientation map of the blood vessels using the bit planes of a gray-scale image. Chakraborti et al. [6] implemented an unsupervised segmentation technique that combines vesselness filter and matched filter using orientation histogram for the segmentation of retinal vessels. Martinez-Perez et al. [7] used a combination of scale space analysis and region growing to segment the vasculature. The technique proposed in [7] was however unable to segment the thin vessels. Zana and Klein [8] used a general vessel segmentation method based on mathematical morphology. However, the technique proposed in [8] was unable to segment the thinner vessels.

Wang et al. [9] proposed multiwavelet kernels and multiscale hierarchical decomposition. Vessels were enhanced using matched filtering with multiwavelet kernels. Szpak and Tapamo [10] used gradient based approach and level set technique. The proposed technique in [10] was however unable to detect the thinner vessels. Vlachos and Dermatas [11] proposed a multiscale retinal vessel segmentation method. The algorithm is based on multiscale line-tracking procedure and morphological postprocessing. However, the proposed technique in [11] was unable to detect the thinner vessels. Mendonça and Campilho [12] combined differential filters for center-line extraction with morphological operators for filling vessel segments considering intensity and morphological properties. Xiao et al. [13] proposed a Bayesian method with spatial constraint for the segmentation of retinal vessels. The spatial dependence of the posterior probability of each pixel in relation to their neighboring pixels was utilized. An energy function was further defined and a modified level set approach was used for the vessel segmentation. Yin et al. [14] implemented a probabilistic tracking-based method for vessel segmentation. A Bayesian method with maximum a posteriori (MAP) was used for detecting the retinal vessel edge points.

Niemeijer et al. [15] proposed pixel classification using a K-nearest neighbour classifier for the segmentation of vessels. Staal et al. [16] proposed a ridge-based vessel segmentation method. The feature vectors were computed for every pixel and classified using a K-nearest neighbour classifier and sequential forward feature selection. Soares et al. [17] generated a feature vector computed from the measurements at different scales of two-dimensional (2D) Gabor wavelet transform on each pixel. Bayesian classifier with Gaussian mixtures was further used to classify the resulting feature space as either vessel or nonvessel pixel. Ricci and Perfetti [18] proposed automated vessel segmentation based on line operators. Two segmentation methods were considered. One of the segmentation methods used two orthogonal line detectors with the gray level of the target pixel to construct a feature vector for supervised classification using a support vector machine. Another segmentation method used by [18] thresholds the response of a basic line detector to obtain unsupervised pixel classification. Marín et al. [19] computed a 7D vector composed of gray-level and moment invariants-based features for pixel representation and used a neural network classifier for the pixel classification. Although the techniques proposed by [15–19] provide high sensitivity and accuracy measures, one major challenge is the requirement of more time for the training phase of the classifiers.

A number of local adaptive thresholding approaches for retinal vessel segmentation such as [20–23] have been proposed in different literatures. Jiang and Mojon [22] proposed an adaptive local thresholding framework using a verification-based multithreshold probing scheme. Although the average running time of the proposed technique in [22] is relatively very fast (8 to 36 seconds), it was unable to detect the thinner vessels. Akram and Khan [20] enhanced the vascular pattern using 2D Gabor wavelet and followed it by a multilayered thresholding technique that applied different threshold values iteratively to generate gray-level segmented image. Cornforth et al. [21] applied wavelet analysis, supervised classifier probabilities, and adaptive threshold procedures, as well as morphology-based techniques. Li et al. [23] combined multiscale analysis based on Gabor filters, scale multiplication, and region-based thresholding to achieve adaptive thresholding for vessel segmentation.

Several other works such as [24–27] have combined pixel thresholding based on certain neighbourhood with global thresholding technique as an adaptive thresholding technique for different segmentation problems.

Gray-level cooccurrence matrix (GLCM) is popularly known for its usage for texture image segmentation [28–31]. Haralick features [28] computed from GLCM have been used for both supervised and unsupervised segmentation. Some known unsupervised gray-level cooccurrence based segmentation techniques have been proposed in some other literatures. Entropy has been one of the few major GLCM features that has often been used for unsupervised segmentation. Different entropy based thresholding such as global, local, joint, and relative entropy has been proposed in [32–36]. Chanwimaluang and Fan [37] proposed the combination of matched filter and entropy for the segmentation of retinal vessels. The performance measure of the proposed technique in [37] was only visual. Li et al. [38] used a threshold selection method based on multiscale edge analysis and gray-level cooccurrence matrix to handle severely degraded document images. A multiscale image description was first used to analyse the image edge; then gray-level cooccurrence matrix was further used to compute the edge pixel pair information. A threshold value was computed using the edge pixel pair cooccurrence matrix. Mokji and Abu Bakar [29] proposed a technique based on the cooccurrence matrix where statistical features were defined from the edge information to handle images that have fuzzy boundaries between the object and the background of the image.

Although much has been achieved in the previous works, the performance measurement and visual results obtained from literatures suggest the need for further research work to address the robust segmentation of both large and thin vessels in a timely efficient manner. This paper proposes a local adaptive thresholding technique using gray-level cooccurrence matrix- (GLCM-) energy information for the robust segmentation of both large and thin vessels in a timely efficient manner.

The rest of this paper is organized as follows.  Section 2 describes the methods and techniques used in this paper.  Section 3 explains the experimental setup and results and discussion, while the conclusion is drawn in Section 4.

## 2. Methods and Techniques

**Figure 1:**
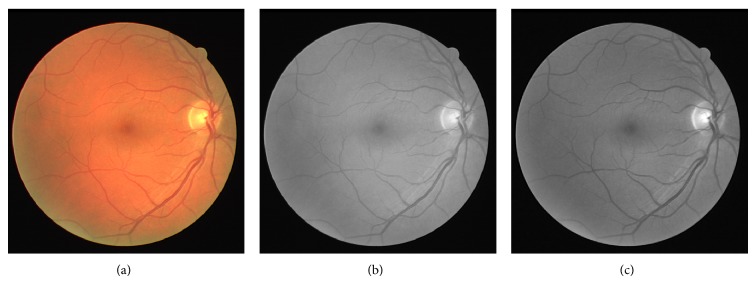
(a) Colored retinal image; (b) grayscale retinal image; (c) Green Channel of the colored retinal image.

The proposed segmentation approach is considered because it utilizes the angular second moment feature which captures the dynamics of the textural information needed for the robust segmentation of both large and thin vessels. A brief description of GLCM and the formulation of a multiscale angular second moment feature matrix are given in this section. This is followed by the proposed local adaptive thresholding technique. Morphological postprocessing technique is finally applied to remove the misclassifications in the segmented vessels. Although most of the previous techniques used the Green Channel of the retinal image, the proposed local adaptive thresholding technique uses the grayscale and the Green Channel of the colored retinal fundus image.

(I) Gray-level cooccurrence matrix: GLCM is usually computed using two key parameters, namely, the relative distance “*d*” between the pixel pair and their relative orientation “Φ.” The distance “*d*” is usually measured in pixel number while “Φ” is quantized in four directions (horizontal: 0°, diagonal: 45°, vertical: 90°, and antidiagonal: 135°). Given a grayscale image *V* of *M* rows and *N* columns, the gray-level cooccurrence matrix *C*(*i*, *j*), for distance “*d*” and direction “Φ,” is defined as
  (1) Ci,j =∑x=0M−1∑y=0N−1PVx,y=i,Vx±dΦ1,y±dΦ2=j,
 where *V*(*x*, *y*) = *i* which means that *i* is the gray-level intensity of pixel (*x*, *y*) and *P* is defined as
  (2) $$\begin{array}{c} P\left(x\right)=\left\{\begin{array}{cc} 1, & \text{if}\;x\;\text{is}\;\text{true},\\ 0, & \text{otherwise}. \end{array}\right. \end{array}$$
 Six features of GLCM were considered by Cataldo et al. [24] to be the most relevant. These features considered are energy, entropy, contrast, variance, correlation, and inverse difference moment. Energy, which is also called angular second moment (ASM) or uniformity, measures the textural uniformity. It is a GLCM feature that detects disorders in textures. Information based on energy feature will be applied for an adaptive thresholding process in this work. The angular second moment is defined as
  (3) $$\begin{array}{c} \text{ASM}=\underset{i}{\sum }\underset{j}{\sum }h{\left(i,j\right)}^{2}, \end{array}$$
 where *h*(*i*, *j*) is the (*i*, *j*)th entry in a normalized gray-tone spatial dependence matrix *C*_(*i*,*j*)_/*R*, with 1/*R* being the normalizing factor.
 The visibility of vessels in a colored fundus retinal image with its grayscale intensity image and the Green Channel of the retinal image are shown in Figure 1. The grayscale intensity and Green Channel of the retinal image are further processed for the segmentation of the retinal vessels.
(II) Multiscale ASM-feature measurement: the variation of energy information within the varying distance “*d*” and relative orientation “Φ” is useful in the design of an adaptive thresholding technique for image segmentation. An ASM-feature matrix across different orientations and distances is computed and defined as
  (4) $$\begin{array}{c} A=\left(a_{ij}\right),\;1\leq i,\;\;j\leq 4, \end{array}$$
 where
  (5) $$\begin{array}{c} a_{ij}={\text{ASM}}_{\left(d_{i},\Phi_{j}\right)},\;1\leq i,\;\;j\leq 4, \end{array}$$
 such that Φ_1_ = 0°, Φ_2_ = 45°, Φ_3_ = 90°, and Φ_4_ = 135°, with distances (*d*_*i*_)_*i*=1,…,4_. The range measure of *A* is given as follows:
  (6) $$\begin{array}{c} {\text{Range}}_{\Phi }=\text{Range}\left(A\right), \end{array}$$
 such that Range_Φ_ is a row vector containing the range of each column of *A*. Three different threshold values are computed from each row vector to segment the retinal vessels. The thresholds from the range measure are
  (7) $$\begin{array}{c} K=0.5\left(\text{MIN}\left({\text{Range}}_{\Phi }\right)\right), \end{array}$$
  (8) $$\begin{array}{c} K=0.5\left(\text{MAX}\left({\text{Range}}_{\Phi }\right)\right), \end{array}$$
  (9) $$\begin{array}{c} K=0.5\left(\text{MEAN}\left({\text{Range}}_{\Phi }\right)\right). \end{array}$$
(III) The proposed local adaptive thresholding technique includes the following.
  (a) Image enhancement is as follows. A combination of unsharp filter, average filter, and contrast enhancement is applied on the grayscale and Green Channel of the retinal image.
  (b) Convolution of the result is obtained in (a) through a median filter using local window size *w*∗*w*. This is described as
    (10) $$\begin{array}{c} U\left(i,j\right)=H\left[x,y\right]*V_{w*w}^{1}\left[x,y\right], \end{array}$$
   where *U*(*i*, *j*) is the convolved retinal image, *V* ^1^[*x*, *y*] is the result obtained in (a) and the convolution mask *H*[*x*, *y*] is a local median filter.
  (c) The difference image *D*(*x*, *y*) is then computed. This is described as
    (11) $$\begin{array}{c} D\left(x,y\right)=U\left(i,j\right)-V^{1}\left[x,y\right]. \end{array}$$
  (d) The segmented image *S*_image_ is obtained as
    (12) $$\begin{array}{c} S_{\text{image}}\left(x,y\right)=\left\{\begin{array}{cc} 0, & \text{if}\;D\left(x,y\right)\leq T\left(x,y\right),\\ 1, & \text{otherwise}, \end{array}\right. \end{array}$$
   where *T*(*x*, *y*) = *K*.
(IV) Postprocessing is as follows. A combination of morphological opening with median filtering process is performed on the inverted thresholded image to handle the remaining misclassifications.

## 3. Experimental Results and Discussion

Experiments were carried out using MATLAB 2010a on an Intel Core i5 2410M CPU, 2.30 GHz, 4 GB of RAM. The proposed method was evaluated using the retinal images on the publicly available DRIVE [39] and STARE [4] databases. DRIVE database is made up of 40 images captured with the use of Canon CR5 camera with 24-bit grayscale resolution and a spatial resolution of 565 × 584 pixels. The 40 images were divided into two. The first group of the DRIVE images is a training set made up of twenty images. The second group is a testing set made up of twenty images. DRIVE database also provides gold standard images as the ground truth for vessel segmentation for the comparative performance evaluation of different vessel segmentation algorithms. STARE database consists of retinal images captured with the use of TopCon TRV-50 fundus camera with 24-bit grayscale resolution and spatial resolution of 700 × 605 pixels. The database provides 20 coloured retinal images and 20 hand-labeled images as the ground truth for the comparative performance evaluation of different vessel segmentation algorithms.

Empirically, we established that window sizes 11 × 11 to 17 × 17 were effective for the segmentation of the retinal vessels. There is however a higher amount of noise and an increase in the computational time when the window size is too large (i.e., larger than 17 × 17). In such a situation, the further postprocessing for removal of noise leads to the removal of the thin vessels as well as some large vessels. This is however caused by the influence of the noneven illumination across the retinal image. In related development, there is a possibility of insufficient data when the window size is too small (i.e., lesser than 11 × 11). This leads to the loss of some large and thin vessels during segmentation. The average time taken for the different window sizes to process each image on DRIVE database ranges from 1.9 to 2.6 seconds.

The performance measures commonly used are sensitivity, specificity, and accuracy. The measures are described in (12)–(14) as follows:

(13) $$\begin{array}{c} \text{Sensitivity}=\frac{\text{TP}}{\left(\text{TP}+\text{FN}\right)}, \end{array}$$

(14) $$\begin{array}{c} \text{Specificity}=\frac{\text{TN}}{\left(\text{TN}+\text{FP}\right)}, \end{array}$$

(15) $$\begin{array}{c} \text{Accuracy}=\frac{\left(\text{TP}+\text{TN}\right)}{\left(\text{TP}+\text{TN}+\text{FP}+\text{FN}\right)}, \end{array}$$

where TP = true positive, TN = true negative, FP = false positive, and FN = false negative.

An event is said to be TP if a pixel is rightly segmented as a vessel and TN when a pixel is rightly segmented as background. In related development, an event is said to be FN if a vessel pixel is segmented to be a background and a FP when a background pixel is segmented as a pixel in the vessel. Sensitivity measure indicates the ability of a segmentation technique to detect the vessel pixels while specificity measure indicates the ability of a segmentation technique to detect background pixels. The accuracy measure indicates the degree of conformity of the segmented retinal image to the ground truth.

A receiver operating characteristic (ROC) curve performance measure is a plot of the rightly classified pixels, referred to as true positive rate (TPR) versus the fraction of the wrongly classified pixels as vessels, referred to as false positive rate (FPR). Area under the curve (AUC) is a performance measure computed from the ROC curve.

The thresholds *K* as computed in (6)–(8) are substituted for *T*(*x*, *y*) in (11) for the segmentation of retinal fundus image.

**Figure 2:**
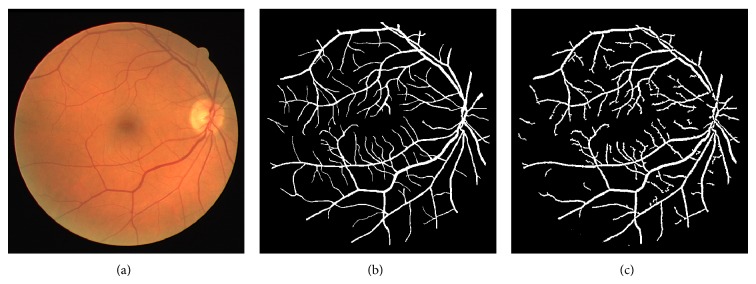
(a) DRIVE colored fundus image; (b) DRIVE database gold standard; (c) segmented vessel of the Green Channel using ASM range information-based threshold value.

**Figure 3:**
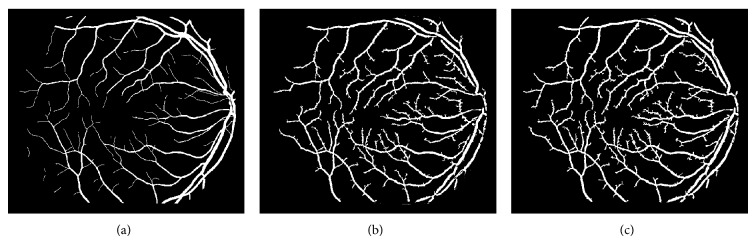
(a) STARE database ground-truth; (b) segmented vessel of the grayscale intensity image using ASM range information-based threshold value; (c) segmented vessel of the Green Channel using ASM range information-based threshold value.

**Figure 4:**
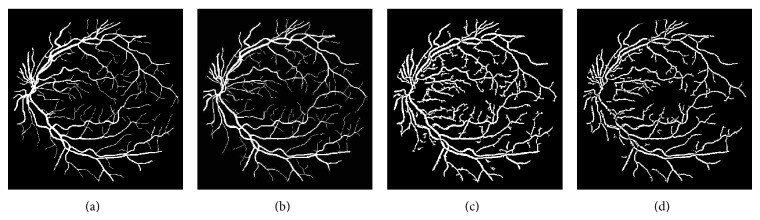
(a) DRIVE database gold standard; (b) manually segmented vessel by the second human observer on DRIVE database; (c) segmented vessel of the Green Channel using ASM range information-based threshold value; (d) segmented vessel of the grayscale intensity image using ASM range information-based threshold value.

**Figure 5:**
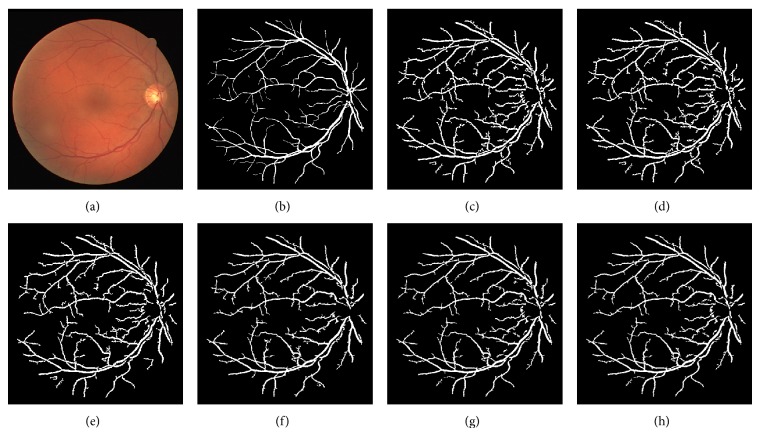
Adaptive thresholding using different ASM range information on DRIVE database. (a) DRIVE database colored retinal image. (b) DRIVE database gold standard. (c) Segmented vessel through adaptive thresholding using minimum ASM mid-range threshold value for the grayscale intensity image. (d) Segmented vessel through adaptive thresholding using maximum ASM mid-range threshold value for the grayscale intensity image. (e) Segmented vessel through adaptive thresholding using mean ASM mid-range threshold value for the grayscale intensity image. (f) Segmented vessel through adaptive thresholding using minimum ASM mid-range threshold value for the Green Channel. (g) Segmented vessel through adaptive thresholding using maximum ASM mid-range threshold value for the Green Channel. (h) Segmented vessel through adaptive thresholding using mean ASM mid-range threshold value for the Green Channel.

The different results obtained from the grayscale intensity image and the Green Channel of the colored fundus image using the proposed ASM-based local adaptive thresholding technique are compared with the manually segmented vessel by the second human observer and the DRIVE database ground truth in Figure 4. Figures 2 and 5 also show the results obtained by the proposed local adaptive thresholding technique based on different ASM range information-based threshold values on DRIVE database. Figures 3 and 10 show the visual results obtained by the proposed local adaptive thresholding technique on STARE database.

Table 1 shows the performance of the different GLCM-energy threshold values for the proposed adaptive thresholding technique using DRIVE database. All the grayscale intensity images have slightly lower sensitivity rates of 0.7397, 0.7313, and 0.7375 than the Green Channel of the colored retinal images with average sensitivity rates of 0.7650, 0.7560, and 0.7632. The grayscale intensity images however have slightly higher accuracies of 0.9488, 0.9511, and 0.9503 over the Green Channel of the colored retinal images with average accuracies of 0.9449, 0.9461, and 0.9477. The maximum mid-range threshold value on the grayscale intensity image yields the best average accuracy rate of 0.9511 while the least average accuracy rate of 0.9449 was achieved using the minimum mid-range threshold value on the Green Channel of the colored retinal images. In related development, the minimum mid-range threshold value on the Green Channel of the colored retinal images yields the highest average sensitivity of 0.7650 while the maximum mid-range threshold value on the grayscale intensity image yields the least average sensitivity of 0.7313. The average specificities of all the grayscale intensity images are slightly higher than the average specificities of the Green Channel of the colored retinal images.

Table 2 shows the performance of the different GLCM-energy threshold values for the proposed adaptive thresholding technique using STARE database. All the grayscale intensity images have slightly lower sensitivity rates of 0.7458, 0.7428, and 0.7427 than the Green Channel of the colored retinal images with average sensitivity rates of 0.7542, 0.7641, and 0.7626. The grayscale intensity images also achieve average accuracy rates of 0.9485, 0.9500, and 0.9504 while the Green Channel of the colored retinal images achieved average accuracy rates of 0.9457, 0.9500, and 0.9510. The average mid-range threshold value on the Green Channel of the retinal image achieved the best average accuracy rate of 0.9510 while the least average accuracy rate of 0.9457 was achieved using the minimum mid-range threshold value on the Green Channel of the colored retinal images.

**Figure 6:**
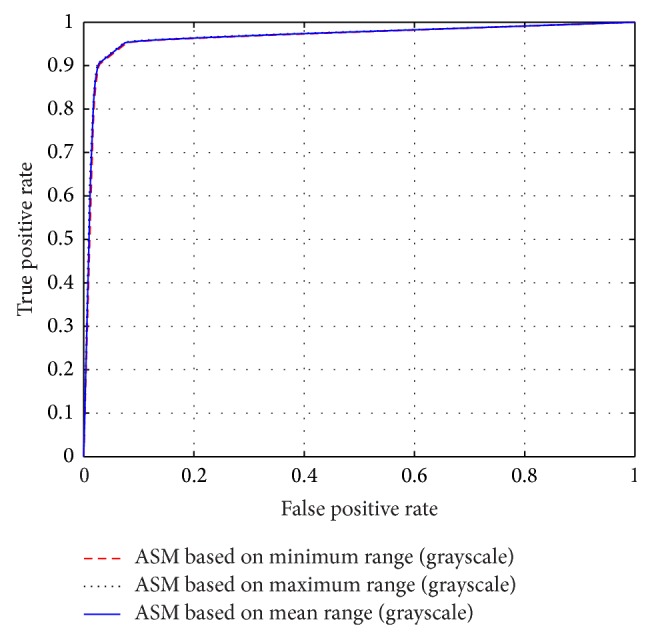
ROC curves showing the performance of each of the adaptive thresholding based on ASM using grayscale on STARE.

**Figure 7:**
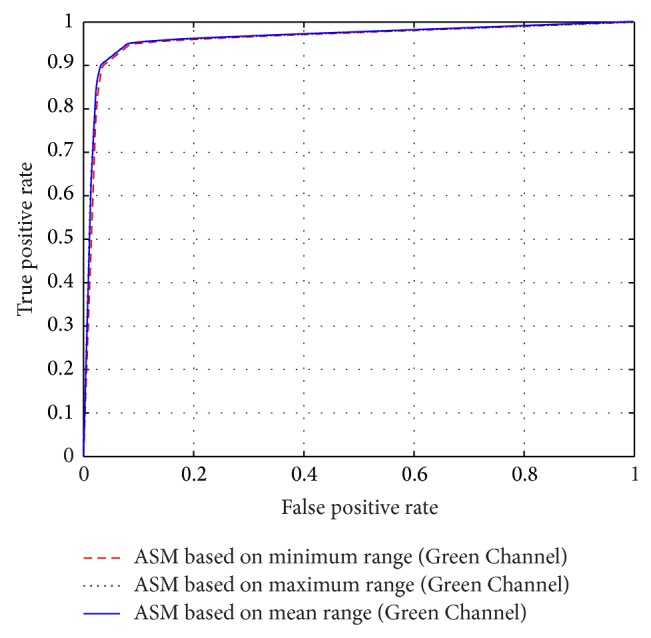
ROC curves showing the performance of each of the adaptive thresholding based on ASM using Green Channel on STARE.

**Figure 8:**
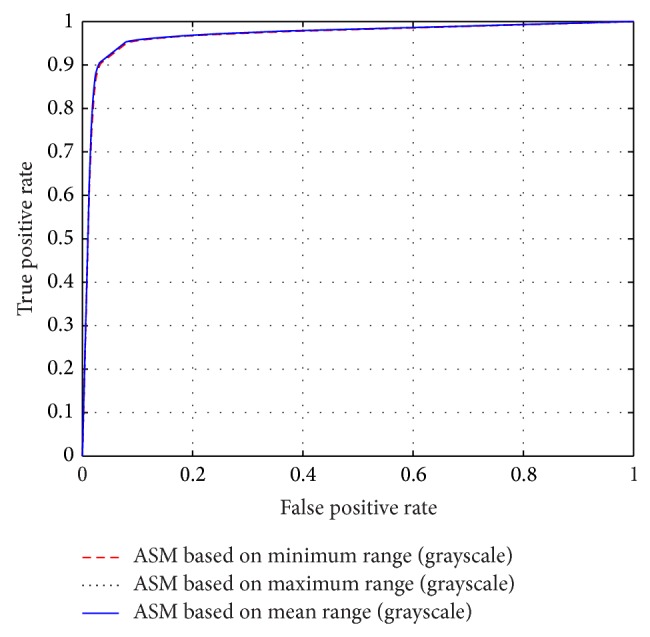
ROC curves showing the performance of each of the adaptive thresholding based on ASM using grayscale on DRIVE.

**Figure 9:**
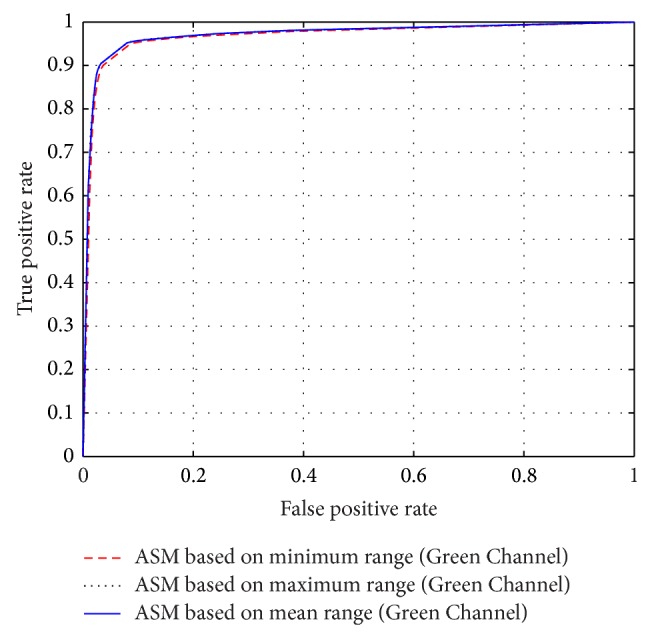
ROC curves showing the performance of each of the adaptive thresholding based on ASM using Green Channel on DRIVE.

**Figure 10:**
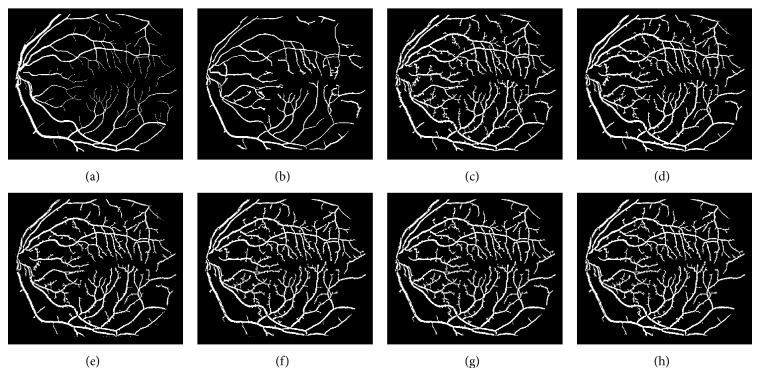
Adaptive thresholding using different ASM range information on STARE database. (a) STARE database ground truth. (b) Segmented retinal vessel by Hoover [4]. (c) Segmented vessel through adaptive thresholding using minimum ASM mid-range threshold value for the grayscale intensity image. (d) Segmented vessel through adaptive thresholding using maximum ASM mid-range threshold value for the grayscale intensity image. (e) Segmented vessel through adaptive thresholding using mean ASM mid-range threshold value for the grayscale intensity image. (f) Segmented vessel through adaptive thresholding using minimum ASM mid-range threshold value for the Green Channel. (g) Segmented vessel through adaptive thresholding using maximum ASM mid-range threshold value for the Green Channel. (h) Segmented vessel through adaptive thresholding using mean ASM mid-range threshold value for the Green Channel.

The performance of the adaptive thresholding based on different ASM range information using grayscale image as depicted through ROC curves is shown in Figures 6 and 8. The ROC curves depicting the performance of the adaptive thresholding based on different ASM range information using Green Channel image are also shown in Figures 7 and 9.

Although all the six thresholds performed well, the slightly higher sensitivity rates achieved by the Green Channel indicate the fact that a bit more vessels were detected when compared to the use of grayscale images. This is because the Green Channel provides the best vessel-background contrast.

In order to compare the performance of the proposed technique with the state of the art, comparison is made with the results obtained by different unsupervised and supervised techniques such as Marín et al. [19], Ricci and Perfetti [18], Soares et al. [17], and Staal et al. [16] as shown in Tables 1 to 4.

### 3.1. Comparison with Existing Segmentation Methods on DRIVE Database

The performance evaluation shows that the works of Chaudhuri et al. [3], Martinez-Perez et al. [7], Vlachos and Dermatas [11], Jiang and Mojon [22], Niemeijer et al. [15], Yin et al. [14], Zana and Klein [8], and Chakraborti et al. [6] present lower average accuracy and lower average sensitivity when compared to all the adaptive thresholding using different ASM range information. Szpak and Tapamo [10] present no average sensitivity but a lower average accuracy when compared to all the adaptive thresholding using different ASM range information. Marín et al. [19] present no average sensitivity but a lower average accuracy when compared to five of the six adaptive thresholding techniques based on ASM information. Soares et al. [17] and Akram and Khan [20] present no average sensitivity but a lower average accuracy when compared to four of the six adaptive thresholding techniques based on ASM information. Mendonça and Campilho [12] present a lower average sensitivity when compared to five of the six proposed thresholds but a lower average accuracy when compared to four of the six thresholds using the investigated techniques.

Staal et al. [16] present a lower average sensitivity when compared to five of the six thresholds but a lower average accuracy when compared to all the threshold values of the investigated technique. Wang et al. [9] present no average sensitivity but a lower average accuracy when compared to four of the six adaptive thresholding techniques based on ASM information. Ricci and Perfetti [18] present no average sensitivity but a higher average accuracy when compared to all the adaptive thresholding techniques based on ASM information. Xiao et al. [13] present a higher average accuracy when compared to all the proposed thresholds but a lower average sensitivity when compared to three of the six proposed thresholds. The average sensitivity of the human observer is higher than all sensitivities of all thresholds of the proposed technique while four of the six average accuracies of the proposed technique are higher when compared to the average accuracy of the human observer.

### 3.2. Comparison with Existing Segmentation Methods on STARE Database

Hoover [4] and Chakraborti et al. [6] present a lower average accuracy and average sensitivity rates when compared to all the adaptive thresholding techniques using different ASM information. The work of Jiang and Mojon [22] also achieves lower average accuracy rate when compared to all the adaptive thresholding using different ASM information. Staal et al. [16] present a higher average accuracy rate when compared to all the average accuracy rates obtained using adaptive thresholding techniques based on ASM information. The average sensitivity rate presented by Staal et al. [16] was however lower when compared to all the average sensitivity rates obtained using adaptive thresholding techniques based on ASM information. Yin et al. [14] present a lower average accuracy rate when compared to all the average accuracy rates obtained using adaptive thresholding techniques based on ASM information.

Mendonça and Campilho [12] and Xiao et al. [13] present lower average accuracy rates when compared to five of the six average accuracy rates obtained using adaptive thresholding techniques based on ASM information. The average sensitivity rates obtained by Mendonça and Campilho [12], Xiao et al. [13], and Yin et al. [14] are lower when compared to all the average accuracy rates obtained using adaptive thresholding techniques based on ASM information. Marín et al. [19], Ricci and Perfetti [18], and Wang et al. [9] present no sensitivity rate but a higher average accuracy rate when compared to all the average accuracy rates obtained using adaptive thresholding techniques based on ASM information. Akram and Khan [20] present no sensitivity rate but a lower average accuracy rate when compared to two of the average accuracy rates obtained using adaptive thresholding techniques based on ASM information. Soares et al. [17] also present no sensitivity but a lower average accuracy rate when compared to five of the average accuracy rates obtained using adaptive thresholding techniques based on ASM information. The average sensitivity of the human observer is higher than all the average sensitivity rates of all the adaptive thresholding techniques based on ASM information. The average accuracy rate of the second observer is however lower when compared to all the average accuracy rates obtained from adaptive thresholding techniques based on ASM information.

All the AUC obtained from the segmentation results achieved by the proposed adaptive thresholding techniques based on ASM information (see Table 3) are higher when compared to the AUC of the previously proposed techniques on DRIVE, while two of the six AUC obtained by the proposed adaptive thresholding techniques based on ASM information on STARE (see Table 4) are higher than the AUC of all the previously proposed techniques.

## 4. Conclusion and Future Work

This paper proposes a local adaptive thresholding technique based on GLCM-energy information for the segmentation of retinal vessels in retinal fundus images. It is shown through different thresholds that the proposed local adaptive thresholding techniques based on energy information perform a robust segmentation from both grayscale intensity and the Green Channel of retinal images. Furthermore, it is shown that the proposed local adaptive thresholding technique is time efficient and gives higher average sensitivity, average accuracy, and AUC values when compared to a wide range of previously proposed techniques on both DRIVE and STARE databases. Future work will investigate the use of soft computing and mainly the introduction of heuristics to detect more thin vessels.

## Figures and Tables

**Table 1:** Performance of different segmentation methods on DRIVE database.

Method	Average accuracy	Average sensitivity	Average specificity
Human observer	0.9473	0.7761	0.9725
Staal et al. [16]	0.9442	0.7345	0.9773
Niemeijer et al. [15]	0.9416	0.7145	0.9801
Zana and Klein [8]	0.9377	0.6971	0.9769
Jiang and Mojon [22]	0.9212	0.6399	0.9625
Vlachos and Dermatas [11]	0.9285	0.7468	0.9551
Wang et al. [9]	0.9461	N/A	N/A
Martinez-Perez et al. [7]	0.9181	0.6389	0.9496
Szpak and Tapamo [10]	0.9299	N/A	N/A
Chaudhuri et al. [3]	0.8773	0.3357	0.9794
Soares et al. [17]	0.9466	N/A	N/A
Akram and Khan [20]	0.9469	N/A	N/A
Mendonça and Campilho [12]	0.9463	0.7315	N/A
Marín et al. [19]	0.9452	0.7067	0.9801
Ricci and Perfetti [18]	0.9595	N/A	N/A
Xiao et al. [13]	0.9529	0.7513	0.9792
Yin et al. [14]	0.9267	0.6522	0.9710
Chakraborti et al. [6]	0.9370	0.7205	0.9579
$\begin{array}{c} K=0.5\left(\text{MIN}\left({\text{Range}}_{\Phi }\right)\right)\\ \left(\text{Gray}\;\;\text{Intensity}\right) \end{array}$	**0.9488**	**0.7397**	**0.9691**
$\begin{array}{c} K=0.5\left(\text{MAX}\left({\text{Range}}_{\Phi }\right)\right)\\ \left(\text{Gray}\;\;\text{Intensity}\right) \end{array}$	**0.9511**	**0.7313**	**0.9724**
$\begin{array}{c} K=0.5\left(\text{MEAN}\left({\text{Range}}_{\Phi }\right)\right)\\ \text{(Gray}\;\;\text{Intensity)} \end{array}$	**0.9503**	**0.7375**	**0.9709**
$\begin{array}{c} K=0.5\left(\text{MIN}\left({\text{Range}}_{\Phi }\right)\right)\\ \left(\text{Green}\;\;\text{Channel}\right) \end{array}$	**0.9449**	**0.7650**	**0.9623**
$\begin{array}{c} K=0.5\left(\text{MAX}\left({\text{Range}}_{\Phi }\right)\right)\\ \left(\text{Green}\;\;\text{Channel}\right) \end{array}$	**0.9477**	**0.7560**	**0.9663**
$\begin{array}{c} K=0.5\left(\text{MEAN}\left({\text{Range}}_{\Phi }\right)\right)\\ \left(\text{Green}\;\;\text{Channel}\right) \end{array}$	**0.9461**	**0.7632**	**0.9634**

**Table 2:** Performance of different segmentation methods on STARE database.

Method	Average accuracy	Average sensitivity	Average specificity
Human observer	0.9354	0.8949	N/A
Hoover [4]	0.9275	0.6751	0.9567
Staal et al. [16]	0.9516	0.6970	N/A
Jiang and Mojon [22]	0.9009	N/A	N/A
Marín et al. [19]	0.9526	0.6944	0.9819
Ricci and Perfetti [18]	0.9584	N/A	N/A
Soares et al. [17]	0.9480	N/A	N/A
Akram and Khan [20]	0.9502	N/A	N/A
Wang et al. [9]	0.9521	N/A	N/A
Mendonça and Campilho [12]	0.9479	0.7123	N/A
Xiao et al. [13]	0.9476	0.7147	0.9735
Yin et al. [14]	0.9412	0.7248	0.9666
Chakraborti et al. [6]	0.9379	0.6786	0.9586
$\begin{array}{c} K=0.5\left(\text{MIN}\left({\text{Range}}_{\Phi }\right)\right)\\ \left(\text{Gray}\;\;\text{Intensity}\right) \end{array}$	**0.9485**	**0.7458**	**0.9649**
$\begin{array}{c} K=0.5\left(\text{MAX}\left({\text{Range}}_{\Phi }\right)\right)\\ \left(\text{Gray}\;\;\text{Intensity}\right) \end{array}$	**0.9500**	**0.7428**	**0.9668**
$\begin{array}{c} K=0.5\left(\text{MEAN}\left({\text{Range}}_{\Phi }\right)\right)\\ \text{(Gray}\;\;\text{Intensity)} \end{array}$	**0.9504**	** 0.7427**	**0.9672**
$\begin{array}{c} K=0.5\left(\text{MIN}\left({\text{Range}}_{\Phi }\right)\right)\\ \left(\text{Green}\;\;\text{Channel}\right) \end{array}$	**0.9457**	**0.7542**	**0.9612**
$\begin{array}{c} K=0.5\left(\text{MAX}\left({\text{Range}}_{\Phi }\right)\right)\\ \left(\text{Green}\;\;\text{Channel}\right) \end{array}$	**0.9500**	**0.7641**	**0.9651**
$\begin{array}{c} K=0.5\left(\text{MEAN}\left({\text{Range}}_{\Phi }\right)\right)\\ \left(\text{Green}\;\;\text{Channel}\right) \end{array}$	**0.9510**	**0.7626**	**0.9657**

**Table 3:** Comparison of AUC of the proposed techniques with previous works on DRIVE.

Method	AUC
Staal et al. [16]	0.9520
Niemeijer et al. [15]	0.9294
Zana and Klein [8]	0.8984
Jiang and Mojon [22]	0.9114
Wang et al. [9]	0.9543
Chaudhuri et al. [3]	0.7878
Soares et al. [17]	0.9614
Akram and Khan [20]	0.963
Marín et al. [19]	0.9588
Ricci and Perfetti [18]	0.9558
Chakraborti et al. [6]	0.9419
ASM_thresh_ = 0.5 (MIN_Range_) (Gray Intensity)	**0.9656**
ASM_thresh_ = 0.5 (MAX_Range_) (Gray Intensity)	**0.9711**
ASM_thresh_ = 0.5 (MEAN_Range_) (Gray Intensity)	**0.9698**
ASM_thresh_ = 0.5 (MIN_Range_) (Green Channel)	**0.9634**
ASM_thresh_ = 0.5 (MAX_Range_) (Green Channel)	**0.9680**
ASM_thresh_ = 0.5 (MEAN_Range_) (Green Channel)	**0.9658**

**Table 4:** Comparison of AUC of the proposed techniques with previous works on STARE.

Method	AUC
Staal et al. [16]	0.9614
Jiang and Mojon [22]	0.929
Wang et al. [9]	0.9682
Soares et al. [17]	0.9671
Akram and Khan [20]	0.970
Marín et al. [19]	0.9769
Ricci and Perfetti [18]	0.9602
ASM_thresh_ = 0.5 (MIN_Range_) (Gray Intensity)	**0.9695**
ASM_thresh_ = 0.5 (MAX_Range_) (Gray Intensity)	**0.9681**
ASM_thresh_ = 0.5 (MEAN_Range_) (Gray Intensity)	**0.9745**
ASM_thresh_ = 0.5 (MIN_Range_) (Green Channel)	**0.9671**
ASM_thresh_ = 0.5 (MAX_Range_) (Green Channel)	**0.9782**
ASM_thresh_ = 0.5 (MEAN_Range_) (Green Channel)	**0.9781**
